# *miR-31* targets ARID1A and enhances the oncogenicity and stemness of head and neck squamous cell carcinoma

**DOI:** 10.18632/oncotarget.11138

**Published:** 2016-08-09

**Authors:** Wen-Cheng Lu, Chung-Ji Liu, Hsi-Feng Tu, Yu-Tung Chung, Cheng-Chieh Yang, Shou-Yen Kao, Kuo-Wei Chang, Shu-Chun Lin

**Affiliations:** ^1^ Institute of Oral Biology, National Yang-Ming University, Taipei, Taiwan; ^2^ Department of Dentistry, National Yang-Ming University, Taipei, Taiwan; ^3^ Department of Dentistry, MacKay Memorial Hospital, Taipei, Taiwan; ^4^ Department of Stomatology, Taipei Veterans General Hospital, Taipei, Taiwan

**Keywords:** ARID1A, cancer, miR-31, stem cell, suppressor

## Abstract

*miR-31* is oncogenic for head and neck squamous cell carcinoma (HNSCC). Proteins containing the AT-rich interacting domain (ARID) modulate the accessibility of chromatin to the transcription machinery needed for gene expression. In this study, we showed that *miR-31* was able to target ARID1A in HNSCC. HNSCC tumors had an inverse *miR-31* and ARID1A expression. *miR-31* associated oncogenicities were rescued by ARID1A expression in HNSCC cells. Furthermore, ARID1A repressed the stemness properties and transcriptional activity of Nanog/OCT4/Sox2/EpCAM via the protein's affinity for AT-rich sites within promoters. HNSCC patients with tumors having high level of *miR-31* expression and high levels of Nanog/OCT4/Sox2/EpCAM expression, together with low level of ARID1A expression, were found to have the worst survival. This study provides novel mechanistic clues demonstrating that *miR-31* inhibits ARID1A and that this enriches the oncogenicity and stemness of HNSCC.

## INTRODUCTION

Head and neck squamous cell carcinoma (HNSCC) arising in the oral cavity, oropharynx, larynx and hypopharynx is the sixth most prevalent malignancy worldwide [1–9]. Several miRNAs, including *miR-21*, *miR-31*, *miR-134* and *miR-196*, have been found to be oncogenic for HNSCC development [1, 4, 7–9]. It is also well known that *miR-31* in other common malignancies, such as lung adenocarcinoma and colorectal carcinoma, is oncogenic [10]. The upregulation of *miR-31* expression in HNSCC is the consequence of EGFR oncogenic activation [5]. Furthermore, *miR-31* could inhibit FIH in turn activating the hypoxia pathway and upregulating VEGF expression [4]. In addition, *miR-31* could also cause an impairment of DNA repair, which leads to an elevated susceptibility of squamous epithelial cells to chemical carcinogenesis [11]. Interestingly, upregulation of *miR-31* is also associated with premalignant disorders that precede the cancerous lesions both in human and murine oral squamous cell carcinoma tissues [2, 12]. The clinical analysis further confirmed that serological *miR-31* could be biomarkers to differentiate HNSCC from the non-cancerous state [13], while a high level of *miR-31* predicted the progression of oral premalignant disorders [14] suggesting that *miR-31* is not only crucial for HNSCC pathogenesis but it also serves as a great diagnostic cue for HNSCC.

The SWI/SNF (switch/sucrose non-fermentable) chromatin-remodeling complex has been shown to activate various cellular processes, including differentiation, proliferation, gene transcription, DNA repair and others [15, 16]. The ARID (AT-Rich Interaction Domain), a major component of the SWI/SNF complex, is a helix-turn-helix motif DNA-binding domain belonging to a family containing 15 highly conserved proteins [17]. ARID1A has been shown to drive adenosine triphosphate (ATP)-dependent helicase activity allowing transcriptional activators and repressors to access onto DNA [16]. Recent studies have demonstrated that ARID1A functions as a tumor suppressor by disrupting aberrant p53 and/or PTEN pathways [18–21]. Loss of ARID1A expression is frequently detected in lung, colorectal, breast, gastric and ovarian clear-cell carcinomas [19, 22–26]. *ARID1A* mutations are found in up to 50% of gynecological tumors, as well as in a broad spectrum of other malignancies [27]. However, the fundamental functions of ARID1A and its downstream effectors during HNSCC have not been fully explored.

Nanog, OCT4, Sox2, KLF4, CD133 and other proteins, which are involved in both protein-protein interaction and transcriptional regulations are factors regulating somatic cell reprogramming [28–30]. Interestingly, SWI/SNF complex has previously been shown to be important to facilitate somatic cell reprogramming [31]. Members of the ARID3 family were found to upregulate the stemness gene expression in human cancers as well as to control pluripotency of hematopoietic stem cells [32–34]. However, the detailed molecular mechanisms involved in regulating the connection between miRNAs, ARID1A and stemness remained to be determined [35, 36]. The present study identifies ARID1A to be a tumor suppressor and a tumor stemness repressor in HNSCC cells. ARID1A trans-inactivates Nanog/OCT4/Sox2 stemness factors as well as Epithelial Cell Adhesion Molecule (EpCAM) [37]. It was also found that the expression profile of the *miR-31*/ARID1A/stemness factors is able to correlate prognosis of patients with HNSCCs. These findings provide new clues as to how *miR-31/* ARID1A enhances oncogenicity and stemness in HNSCC cells.

## RESULTS

### Decreased ARID1A expression in HNSCC tissues

**Figure 1:**
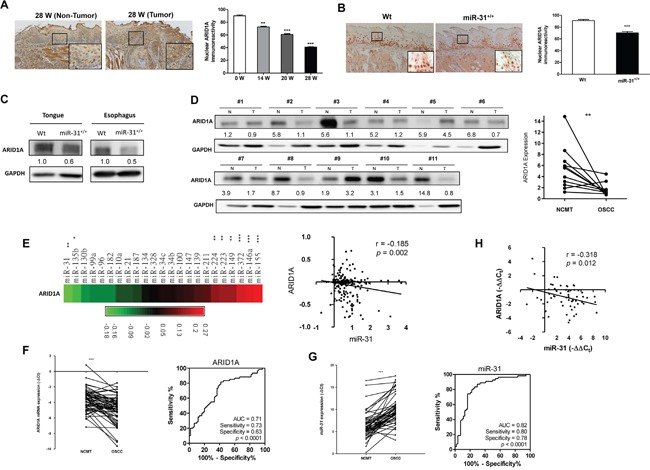
Decreased ARID1A expression in HNSCC **A.** Lt, Representative ARID1A immunohistochemical analysis of the non-tumor (Lt) epithelium and tumor samples (Rt) from the tongues in mice following 4NQO induction for 28 weeks. (x100); Indents, higher power views of blocks (x400). Rt, The quantification shows a progressive downregulation of nuclear ARID1A immunoreactivity in the tongue epithelium during 4NQO-induced mouse tongue carcinogenesis. **B.** Lt, Representative ARID1A immunohistochemistry of the tongue epithelium of Wt (Lt) and *miR-31^+/+^* transgenic mice. (x100); Indents, higher power view of the blocks. (x400). Rt, Quantification of nuclear ARID1A immunoreactivity. Downregulation of nuclear ARID1A expression in the tongue epithelium of *miR-31^+/+^* transgenic mice can be seen. **C.** Western blot analysis of the tongue and esophageal epithelium stripped from mice. This shows the downregulation of ARID1A in both the tongue and esophageal epithelium of *miR-31^+/+^* transgenic mice relative to Wt mice. **D.** Lt, Western blot analysis of ARID1A protein expression in 11 human HNSCC tissue pairs. N, NCMT; T, HNSCC. Rt, Quantitation. Numbers below the Western blot pictures are the normalized values. **E.** Linear regression analysis to correlate ARID1A expression and the expression of oncogenic miRNAs using HNSCC TCGA database. Lt, Algorithm of *r* values. Rt, An inverse correlation is noted between *miR-31* and ARID1A. **F.** Lt, qRT-PCR analysis of *ARID1A* mRNA expression in 58 HNSCC/NCMT tissue pairs. Rt, ROC analysis. AUC: area under curve. **G.** Lt, qRT-PCR analysis of *miR-31* expression in tissue pairs. Rt, ROC analysis. **H.** Linear regression analysis shows an inverse correlation between *miR-31* expression and *ARID1A* mRNA expression in the tissue pairs.

Our recent report has demonstrated that numbers of oncogenic miRNAs could be induced in 4-Nitroquinoline 1-Oxide (4NQO) treated mice [12]. By taking advantage of this model, it was found that nuclear ARID1A expression was progressively decreased in the tongue epithelium of mice with long-term 4NQO treatment (Figure 1A, Supplementary Figure S1). Furthermore, a significant downregulation of nuclear ARID1A expression was also detected in the neoplastic tongue and esophagus tissues in a previously established K14-EGFP-*miR-31* transgenic mouse compared to control mouse tissues [11] (Figure 1B, 1C). We then investigated ARID1A protein expression in HNSCC tissue pairs. ARID1A protein expression was found to be lower in tumor tissue samples compared to paired non-cancerous matched tissues (NCMTs) (Figure 1D). Next we retrieved the expression profiles of 23 oncogenic miRNAs from The Cancer Genome Atlas (TCGA) database for HNSCC and correlated these with *ARID1A* expression [38]. The results indicated that *miR-31* and *miR-135b* level was negatively correlated with *ARID1A* expression (Figure 1E, Supplementary Figure S2). Analysis of 58 tissue pairs demonstrated an obvious decrease in *ARID1A* mRNA in tumors relative to their normal counterparts as ROC curves indicated a predictive power of 0.71 for the separation of non-diseased samples from HNSCC samples (Figure 1F). The quantitative RT-PCR analysis also confirmed a significant upregulation of *miR-31* in HNSCC with a ROC value as high as 0.82 when distinguishing benign tissue from malignant tissue (Figure 1G). An inverse correlation between the expression of *ARID1A* and *miR-31* was noted in our study cohort (Figure 1H) revealing a potential relationship between *miR-31* level and ARID1A expression in HNSCC tissues.

### *miR-31*/ARID1A axis drives HNSCC tumorigenesis

**Figure 2:**
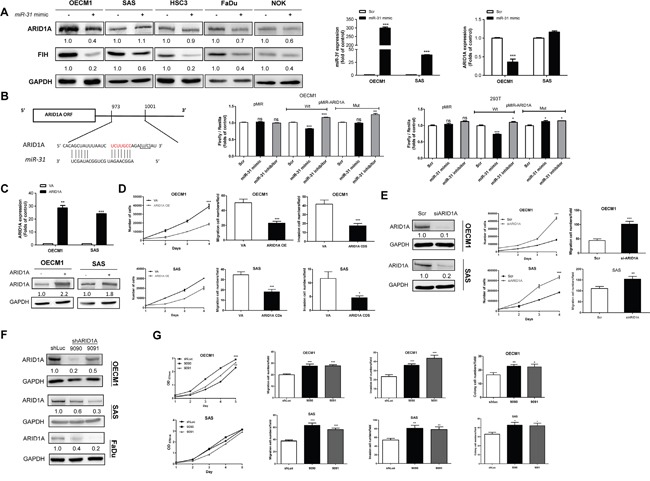
*miR-31* inhibits ARID1A in HNSCC **A.** Lt, Ectopic *miR-31* expression downregulates ARID1A expression in the OECM1, HSC3, FaDu and NOK cell lines, but not in the SAS cell line. Middle and Rt, qRT-PCR analysis targeting *miR-31* and *ARID1A* mRNA expression, respectively, in OECM1 and SAS cell. **B.** Lt, Similarity between *miR-31* and the 3′UTR sequence of the *ARID1A*. Rt, Reporter assays. Lt, Assays of Wt and Mut reporters after treatment with *miR-31* mimic or *miR-31* inhibitor in OECM1 and 293T cells. **C.** Ectopic ARID1A expression in OECM1 and SAS cells. Upper, mRNA expression; Lower, protein expression. **D.** Phenotypic analysis of the OECM1 (Upper) and SAS (Lower) cells. Lt, proliferation; Middle, migration; Rt, invasion. **E.** Knockdown of ARID1A protein expression in OECM1 and SAS cells using the siARID1A oligonucleotide. Lt, protein expression; Middle, proliferation; Rt, migration. **F.** Establishment of OECM1, SAS and FaDu cell subclones with ARID1A knockdown. **G.** Phenotypic analysis of the OECM1 (Upper) and SAS (Lower) cell subclones. Lt to Rt, proliferation, migration, invasion and AIG. Numbers below the Western blot pictures are the normalized values.

Ten different *in silico* modules unequivocally predicts that the 3′ untranslated region (3′UTR) of the ARID1A transcript contains a binding site for *miR-31* (Supplementary Figure S3). But the direct targeting of *miR-135b* to ARID1A transcript seems unlikely. We then determined whether *miR-31* directly controls ARID1A via post-transcriptional regulation. Upon the treatment of a *miR-31* mimic, ARID1A expression was downregulated in most HNSCC cells. qRT-PCR further confirmed that an increased *miR-31* expression was associated with a decreased *ARID1A* mRNA expression in OECM1 cells, but not in SAS cells (Figure 2A). In order to determine whether ARID1A transcript is a direct target of *miR-31*, we constructed wild-type (Wt) and mutant (Mut) reporter plasmids (Figure 2B) for a reporter assay. The results indicate that *miR-31* is able to repress the luciferase activity of the Wt reporter, but not in Mut form, in OECM1 and 293T cells under *miR-31* mimic transfection (Figure 2B). This cell-type variance could possibly be explained by a T→C polymorphism at rs12685 within the ARID1A 3′UTR in SAS cells, but not in other cells (Supplementary Figure S4A). To further clarify if this polymorphism reduces the affinity between *miR-31* and the potential binding sequence, we generate the single nucleotide polymorphism (SNP) reporter from the Wt reporter in which the T was replaced by C at rs12685. The reporter assay indicated that the *miR-31* suppressed Wt reporter activity was abrogated in SNP reporter in both OECM1 and 293T cells (Supplementary Figure S4B). It is worth noting that 32 clinical HNSCC samples were all wild type at rs12685 indicating that the polymorphism found in SAS cells is rather infrequent in human HNSCC tissues. In summary, these findings confirmed that *miR-31* directly inhibits ARID1A through its 3′UTR region, and that rs12685 is able to interfere with *miR-31* targeting.

### ARID1A suppresses the oncogenicity of HNSCC cells

**Figure 3:**
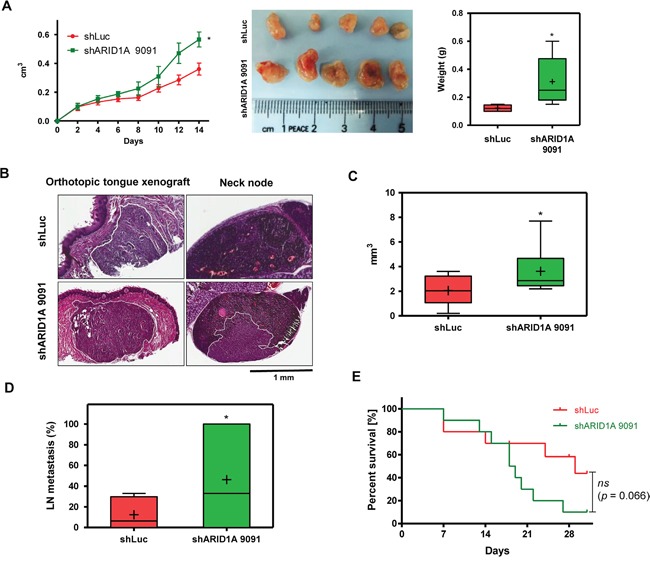
Knockdown of ARID1A expression increases xenografic tumorigenesis and neck metastasis **A.** Subcutaneous xenografic tumorigenesis of SAS cell subclones (*n* = 5). Lt, Growth curve; Middle, tumors harvested; Rt, weight of tumors. **B-E.** Orthotopic xenografic tumorigenesis of SAS cell subclones (*n* = 10). (B) H&E-stained tissue sections. Lt, tongue tissues, Rt neck nodes. The primary tumors in tongue and metastatic lesion in neck node are marked with white solid lines. (C) Volume of primary tumors. (D) Percentage of neck metastasis in tumors < 6 mm^3^ in size. (E) Survival curve of mice. Bar charts, Box and Whiskers plot. Line, medium value; +, mean value.

A number of cellular assays were performed to define the regulatory role of ARID1A for malignancy in HNSCC cells. Overexpression of ARID1A resulted in a decreased proliferation, migration and invasion in OECM1 and SAS cells (Figure 2C, 2D). In contrast, downregulation of ARID1A expression led to an increase in proliferation, migration and colony formation ability in different HNSCC cells compared to control cells *in vitro* (Figure 2E-2G). However, endogenous expression of *miR-31* (Supplementary Figure S5A, Lt) and ARID1A (Supplementary Figure S5A, Rt) were not correlated with migration ability (Supplementary Figure S5B) in HNSCC cell lines tested (Supplementary Figure S5C). *In vivo* models were thus used to validate the oncogenic enrichment associated with ARID1A downregulation using SAS cell subclones. An enhanced tumor growth associated with ARID1A downregulation was noted in subcutaneous xenograft model (Figure 3A). In orthotopic tongue xenograft model, downregulation of ARID1A expression was found associated with a higher induction of primary tumorigenesis (Figure 3B, 3C), a higher percentage of nodal metastasis (Figure 3B, 3D), and a trend of worse survival of hosted mice (Figure 3E).

### *miR-31* upregulate stemness markers through ARID1A inhibition

**Figure 4:**
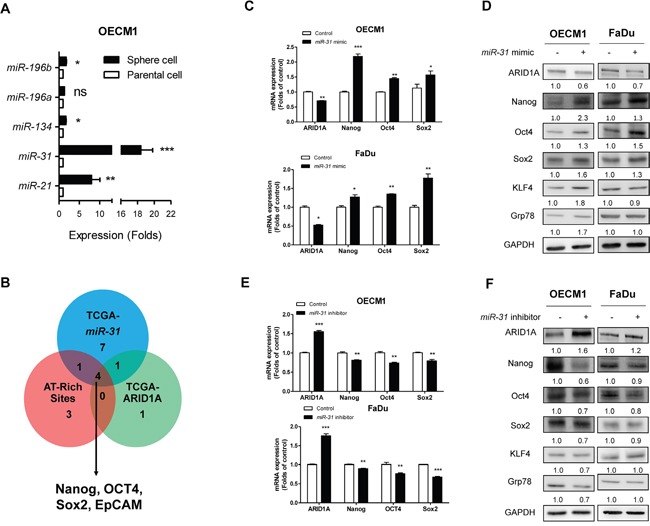
*miR-31* downregulates ARID1A and upregulates pluripotency genes **A.** qRT-PCR analysis of *miR-21*, *miR-31*, *miR-134*, *miR-196a* and *miR-196b* in OECM1 parental cells and OECM1 sphere cells. A significant increase in *miR-31* expression which is much greater than other miRNAs, is found in the sphere cells. **B.** Bioinformatics analysis indicates that expression levels of Nanog, OCT4, Sox2 and EpCAM genes are positively correlated with the level of *miR-31* expression, but are negatively correlated with the level of ARID1A expression; this is identified using the HNSCC-TCGA database. These genes contain predicted AT-rich sites in their promoters. **C, D.** Treatment of OECM1 and FaDu cells with *miR-31* mimic. This downregulates ARID1A protein expression and upregulates Nanog/OCT4/Sox2 protein expression (in C). The treatment also downregulates *ARID1A* mRNA expression and upregulates *Nanog/OCT4/Sox2* mRNA expression (in D) in both OECM1 and FaDu cells. **E, F.** Treatment with *miR-31* inhibitor. This upregulates ARID1A and downregulates Nanog/OCT4/Sox2 in protein expression (in E) and mRNA expression (in F) in both OECM1 and FaDu cells. It should be noted that the protein expression levels of KLF4 and Grp78 are upregulated by *miR-31* mimic and downregulated by *miR-31* inhibitor in OECM1 cells. Numbers below Western blot pictures are the normalized values.

To explore potential stemness associated effectors controlled by the *miR-31*/ARID1A pathway, sphere cell population of OECM1 cell was analyzed to show conspicuous increase of *miR-31* expression (Figure 4A). In addition, the correlation of 22 stemness genes with *miR-31* expression was analyzed using the TCGA database to show that thirteen genes were positively correlated with *miR-31* expression (Supplementary Figure S6) and six genes were negatively correlated with *ARID1A* (Supplementary Figure S7). Among them, five genes were overlapped. Using a Genomatrix analytical module, an appropriate AT-rich site was found in the promoter regions of genes Nanog, OCT4, Sox2 and EpCAM (Supplementary Figure S8), suggesting that these genes could possibly be *miR-31*-ARID1A downstream genes (Figure 4B). To further validate the role of *miR-31* in modulating the stemness properties of HNSCC cells, the Nanog/OCT4/Sox2 protein expression was determined in OECM1 and FaDu cells treated with *miR-31* mimic or *miR-31* inhibitor. The results showed that *miR-31* downregulated ARID1A expression and upregulated Nanog/OCT4/Sox2 mRNA and protein expression in both cells (Figure 4C, 4D). On the contrary, *miR-31* inhibition upregulated ARID1A expression and downregulated Nanog/OCT4/Sox2 mRNA and protein expression (Figure 4E, 4F). These finding suggested a potential interplay between *miR-31* and ARID1A to induce HNSCC stemness related genes expression.

### ARID1A represses pluripotency genes and suppresses stemness properties

**Figure 5:**
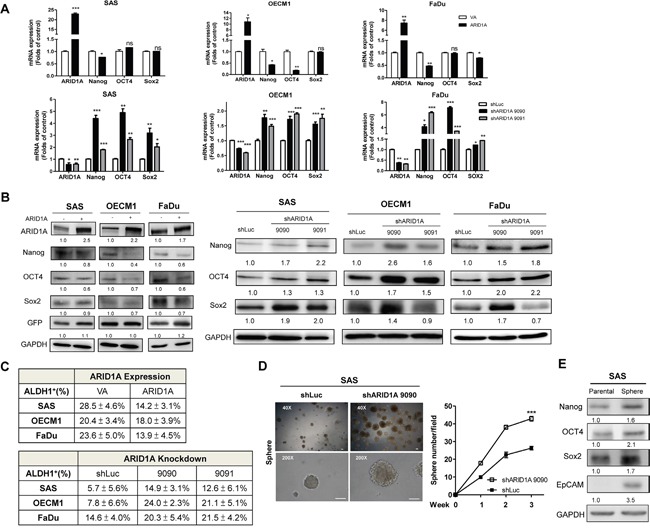
ARID1A downregulates pluripotency genes and stemness **A.** qRT-PCR analysis and **B.** Western blot analysis of ARID1A, Nanog, OCT4 and Sox2 expression in the SAS, OECM1 and FaDu cell subclones with ARID1A overexpression (A, Upper; B, Lt) and knockdown (A, Lower; B, Rt). **C.** Summary of ALDH1^+^ cell population of ARID1A overexpression or knockdown in HNSCC cells. **D.** Spheroid formation assay by SAS cells. Lt, Images at the 3^rd^ week; Rt, Quantification of spheres greater than 100 μm in size at week 1-3. Bars, 100 μm. The shARID1A 9090 SAS cell subclone shows a higher capability for sphere formation relative to the control. **E.** Western blot analysis. This shows that there is upregulation of Nanog/OCT4/Sox2/EpCAM in spheres derived from SAS cells compared to parental SAS cell. Numbers below the Western blot pictures are the normalized values.

HNSCC cells with exogenous ARID1A expression were shown to have lower level of Nanog/OCT4/Sox2 mRNA and protein expression (Figure 5A, Upper, Figure 5B, Lt). In contrast, Nanog/OCT4/Sox2 expression was found to be increased in the ARID1A deficient cells (Figure 5A, Lower; Figure 5B, Rt). Aldehyde dehydrogenase (ALDH1^+^) expression was reported to be a putative stemness maker in various cancers [39]. A decrease in ALDH1^+^ activity was shown in HNSCC cells with ARID1A overexpression compared to control ones; ARID1A deficiency, in contrast, led to a higher percentage of ALDH1^+^ HNSCC cells (Figure 5C, Supplementary Figure S9). The spheroid-forming capability in HNSCC cells with ARID1A knockdowned cells was also examined. The results showed that ARID1A expression could possibly inhibit sphere formation in HNSCC cells (Figure 5D). Interestingly, a drastic increase of Nanog/OCT4/Sox2/EpCAM expression was noted in SAS sphere cells (Figure 5E). Collectively, these results suggest that ARID1A inhibits oncogenesis and stemness properties in HNSCC cells by downregulating stemness factors.

### ARID1A repressed EpCAM expression

**Figure 6:**
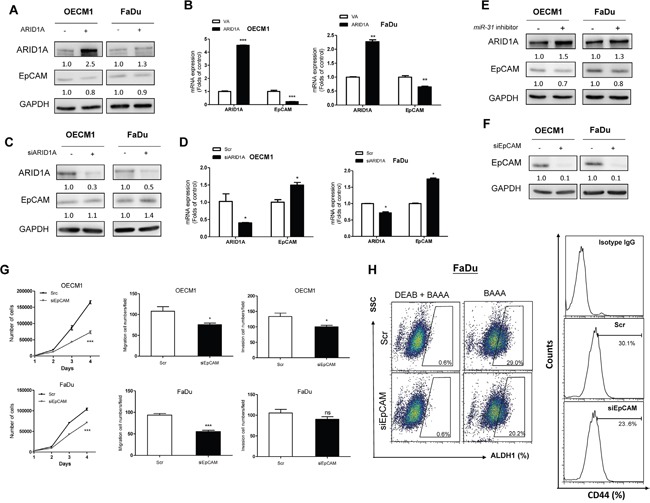
ARID1A-EpCAM is involved in oncogenicity and stemness of HNSCC cells **A-G.** OECM1 and FaDu cells. (A, B) Ectopic ARID1A expression, (C, D) Knockdown of ARID1A expression. (A, C) Western blot analysis. (B, D) qRT-PCR analysis. An inverse relationship between the expression level of ARID1A and EpCAM should be noted. (E) Ectopic *miR-31* expression. This results in ARID1A upregulation and EpCAM downregulation. (F) Treatment with siEpCAM oligonucleotide. This downregulates EpCAM protein expression. (G) Upper, OECM1 cell; Lower, FaDu cell. Analysis of proliferation (Lt), migration (Middle) and invasion (Rt). **H.** FaDu cell. Knockdown of EpCAM decreases the ALDH1^+^ (Lt) and CD44^+^ (Rt) cell populations. The numbers below the Western blot pictures are the normalized values.

To ascertain whether *miR-31*-ARID1A-EpCAM also involved in oncogenicity and stemness, we expressed or knocked down ARID1A in OECM1 and FaDu cells. An inverse correlation between the expression of ARID1A and the expression of EpCAM at both the protein level (Figure 6A, 6C) and mRNA level (Figure 6B, 6D) was noted. In addition, *miR-31* inhibition was able to upregulate ARID1A, but downregulated EpCAM in both types of cells (Figure 6E). Knockdown of EpCAM using siRNA decreased the oncogenic phenotypes (Figure 6F; Figure 6G, Upper) of OECM1 cells. EpCAM knockdown in FaDu cells significantly decreased the growth and migration of these cells, but only slightly decreased invasion by these cells (Figure 6F; Figure 6G, Lower). EpCAM knockdown in FaDu cells also decreased the ALDH1^+^ (Figure 6H, Lt) and CD44^+^ (Figure 6H, Rt) cell populations.

### ARID1A trans-inactivates Nanog/OCT4/Sox2/EpCAM and rescues *miR-31* functions

**Figure 7:**
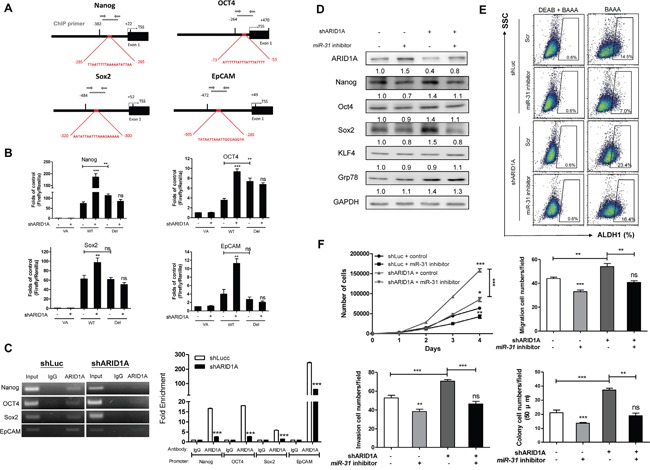
*miR-31* inhibits ARID1A to transactivate pluripotency genes and oncogenicity **A.** Schematic diagrams designate AT-rich sites (Red boxes) in the proximal regions of the Nanog, OCT4, Sox2 and EpCAM promoters. TSS, transcription start site; Thin black bars, the termini of AT-rich sites. Thick black bars define the segments for reporter assay in **B.** Grey lines define the segments for ChIP assay in **C.** (B) Promoter activity. Knockdown of ARID1A increases the activity of the WT promoter reporters of each gene, while it does not alter the activity of the Del promoter reporters. Deletion of the AT-rich sites increases the promoter reporter activity of Nanog and OCT4, but it does not alter the promoter reporter activity of Sox2 and EpCAM. (C) Lt, ChIP assays of ARID1A in Nanog/OCT4/Sox2/EpCAM promoter regions. Knockdown of ARID1A reduces the PCR products amplified from the DNA fragments that had been immunoprecipitated by anti-ARID1A antibody. Rt, ChIP qPCR analysis. Input, 2% of total lysate. **D-F.** Rescue of oncogenicity and stemness in the OECM1 shARID1A 9091 cell subclone and control. (D) Upregulation of Nanog, OCT4 and Sox2, induced by ARID1A knockdown, is reversed by *miR-31* inhibition. This does not occur with KLF4 or Grp78. (E) An increase in the ALDH1^+^ cell population induced by ARID1A knockdown is also reversed by *miR-31* inhibition. (F) The proliferation (Upper Lt), migration (Upper Rt), invasion (Lower Lt) and AIG (Lower Rt) are induced by ARID1A knockdown and this is rescued by *miR-31* inhibition. Numbers below the Western blot pictures are the normalized values.

Figure 7A and Supplementary Figure S10 illustrate the predicted AT-sites in the proximal promoter regions of Nanog/OCT4/Sox2/EpCAM. To clarify whether ARID1A directly downregulated expression of these genes, promoter-driven luciferase reporter constructs, either containing a wild type full-length promoter sequence (designated WT), or containing a deleted promoter sequence with truncation at the AT-rich site (designated Del) were created. In the OECM1 shLuc cell subclone, the WT promoters and Del promoter had higher activities compared to the VA control. In addition, the activity of Del was higher than that of WT in Nanog and OCT4 promoters. The activity levels of the WT promoters in shARID1A 9090 cell subclone were much higher than those of the shLuc cell subclone. However, the activity of Del promoter between shARID1A 9090 cell subclone and shLuc cell subclone was not different (Figure 7B). A chromatin immunoprecipitation (ChIP) assay was performed to detect the binding of ARID1A to the AT-rich sites of the Nanog/OCT4/Sox2/EpCAM promoters using the OECM1 cell subclones. Amplification of the immunoprecipitates created by the anti-ARID1A antibody showed that the presence of the Nanog/OCT4/Sox2/EpCAM fragments that contained the AT-rich sites using the shLuc cell subclone. The intensity of the PCR products was found to be reduced in the OECM1 shARID1A cell subclone (Figure 7C, Lt). Quantitation showed drastic enhancement of ARID1A binding at the AT-rich site of the Nanog/OCT4/Sox2/EpCAM promoter regions compared to the IgG control (Figure 7C, Rt). This binding was attenuated by knockdown of ARID1A. These findings demonstrate that ARID1A represses Nanog/OCT4/Sox2/EpCAM expression directly by binding to their AT-rich sites, which are found within the proximal promoter region.

The repression of Nanog/OCT4/Sox2 expression results from *miR-31* inhibition was rescued by the knockdown of ARID1A in OECM1 cells (Figure 7D). The reduction in the ALDH1^+^ cell population meditated by *miR-31* inhibition was ameliorated by the knockdown of ARID1A (Figure 7E). The suppression of oncogenicity as a result of *miR-31* inhibition was also rescued by the knockdown of ARID1A expression (Figure 7F). The *miR-31*-ARID1A axis controls HNSCC stemness and oncogenicity.

### Inverse expression relationship between *miR-31* and ARID1A in human HNSCC tissue samples

**Figure 8:**
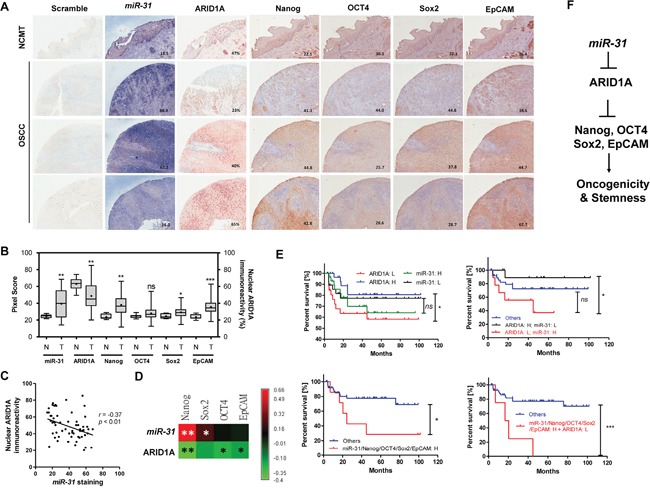
ISH and IHC analysis using HNSCC TMA tissue samples **A.** NCMT (Upper most) and 3 representative HNSCC TMA tissue cores. (x100). **B.** Quantification of *miR-31*, nuclear ARID1A, Nanog, OCT4, Sox2 and EpCAM immunoreactivities in NCMT and HNSCC tissue cores. **C.** An inverse correlation can be seen between *miR-31* staining and nuclear ARID1A immunoreactivity. **D.** The algorithm of *r* values demonstrates a positive correlation between *miR-31* staining and the expression of stemness factors together with an inverse correlation between ARID1A expression and the expression of stemness factors. **E.** Kaplan-Meier survival analysis used to assess the disease free survival of HNSCC patients. H, high expression; L, low expression. **F.** Illustration of *miR-31*-ARID1A-Nanog/OCT4/Sox2/EpCAM axis.

*In situ* hybridization (ISH) and immunohistochemistry (IHC) analyses were performed on an HNSCC tissue microarray (TMA) containing 60 tumors and some NCMT tissue cores. *miR-31* staining (blue pixels) along with ARID1A immunoreactivity (nuclear percentage) and Nanog/OCT4/Sox2/EpCAM immunoreactivities (brown pixels) were scored (Figure 8A). An increase in *miR-31* expression and a decrease in nuclear ARID1A expression from NCMT to HNSCC was noted (Figure 8B). Higher Nanog/Sox2/EpCAM expression in tumors was also noted. A significant inverse correlation between *miR-31* expression and ARID1A expression was found using the HNSCC tumor samples (Figure 8C). Moreover, Nanog and Sox2 expression was positively correlated with *miR-31* expression, and Nanog, OCT4 and EpCAM expression was inversely correlated with ARID1A expression (Figure 8D). The staining was classified from low to high using the median values of percentages or the pixel scores as the cutoff. Kaplan-Meier analysis indicated an association between a lower level of ARID1A expression and a poorer disease-free survival by the patient (Figure 8E, Upper Lt). Tumors that had a high level of *miR-31* expression and a low level of ARID1A expression exhibited a much worse patient prognosis than tumors carrying the other possible expression profiles (Figure 8E, Upper Rt). Tumors with a higher expression level of individual pluripotency gene, with a higher co-expression level of all pluripotency genes (Supplementary Figure S11) or with a higher expression level of *miR-31* (Figure 8E, Upper Lt) did not display a lower survival. However, tumors carrying a higher level of expression of pluripotency genes together with a higher level of expression of *miR-31* were found to have a poorer prognosis than the other patterns (Figure 8E, Lower Lt). Finally, tumors carrying a high level of expression of *miR-31*, a higher level of expression of pluripotency genes and lower level of ARID1A expression were found to have a very poor survival (Figure 8E, Lower Rt). In summary, this study specifies the *miR-31-* ARID1A-stemness genes regulatory axis in HNSCC pathogenesis (Figure 8F).

## DISCUSSION

*miR-31* is significantly upregulated during HNSCC and this miRNA induces hypoxia and epithelial-mesenchymal transition, and disrupts gene repair by inhibiting FIH, Ku80, Parp1 and various other targets during tumorigenesis [2, 4, 11]. Moreover, *miR-31* is upregulated by EGF and this occurs early in HNSCC tumorigenesis [2, 5, 12, 14]. This study has further indicated that *miR-31* inhibits ARID1A in HNSCC [36]. Previous studies have reported that *miR-200c*, *miR-34*, *Let7* and *Lin28* are involved in cancer stemness [32, 33, 40, 41]. This study provides a new line of evidence showing that *miR-31* upregulates various pluripotency genes and increases the stem cell population in HNSCC [42]. Since ARID1A also represses stemness, the suppressor activity of ARID1A relative to other *miR-31* targets is being investigated.

We have identified in the present study that a low nuclear expression of ARID1A predicts a worse HNSCC patient survival. Mice carrying xenografic tumors with low ARID1A expression have more extensive metastasis and worse survival. Despite the fact that ARID1A is known to affect p53-p21 and AKT in order to suppress oncogenicity [18-20, 43], this study further shows that ARID1A trans-inactivates the Nanog/OCT4/Sox2 stemness genes; these are known, together with EpCAM tumor antigen, to be involved in the repression of both oncogenesis and stemness in HNSCC. As the ARID3 family complex also promotes the stemness properties of HNSCC [32, 33], the regulation of the various subunits that form the SWI/SNF complex and drive tumor stemness needs to be further investigated.

Since an inverse association in expression between *miR-31* expression and ARID1A expression was found to occur both in our study cohort and via the TCGA database, high expression of *miR-31* could be a crucial mechanism with respect to ARID1A downregulation in HNSCC. It is thus likely that *miR-31* overexpression, in addition to deletion, mutation or epigenetic disruption of this gene, may underlie ARID1A downregulation in both lung and colorectal carcinomas [24, 26]. Our preliminary findings also suggest that a T→C polymorphism at rs12685, which is adjacent to the *miR-31* binding site within the 3′UTR of ARID1A, is able to confound the binding of *miR-31* onto ARID1A. However, due to the low frequency of occurrence of this polymorphism in the human population, the importance of this to the interaction of *miR-31*-ARID1A during cancer is likely to be limited.

This study indicates that ARID1A transcriptionally represses Nanog/OCT4/Sox2 concomitantly by binding to the AT-rich sites present in their promoters. ARID1A knockdown increases wild-type promoter activity, while in parallel experiments ARIDIA is unable to alter the activity of promoters lacking the appropriate AT-rich sites. However, since the truncation of the AT-rich sites in Sox2 and EpCAM does not increase the endogenous activity of the reporter genes, it seems that such truncation may still show a potential for repression via a new alignment of sequences created by the deletion. Although studies have shown the prognostic implications of Nanog/OCT4/Sox2 to HNSCC [30, 44, 45], this study only identified a trend towards worse survival among HNSCC patients when they had high expression of these stemness factors.

The EpCAM membranous molecule is a tumor marker widely used to capture circulatory tumor cells [46]. It is also a potential marker for tumor stemness in hepatocellular carcinoma [47]. This study provides clues indicating the modulation of EpCAM and the effect this has on oncogenicity and stemness may be important during HNSCC. Since *miR-31*-ARID1A upregulates the expression of Nanog/OCT4/Sox2/EpCAM, together with the fact that the activation of this axis creates a worst HNSCC prognosis, a strategy that reduces the effects of this axis may be a useful therapeutic approach when trying to address HNSCC.

## MATERIALS AND METHODS

### Tissue samples

The surgical specimens consisted of primary tumors along with paired NCMTs and were collected for qRT-PCR analysis and Western blot analysis (Supplementary Table S1). In addition, HNSCC TMAs consisting of HNSCC tumor cores and some paired HNSCC/NCMT cores (Supplementary Table S2) were also investigated [5]. This study was approved by the Institutional Review Board (approval number 13MMHIS274). Archival murine tongue, esophagus, and tumor tissues were obtained during our previous studies [11, 12].

### Quantitative (q)RT-PCR

Total RNAs were reversely transcribed into cDNA. The levels of expression of various genes were analyzed using the TaqMan Assay system according to the manufacturer's instructions (Applied Biosystems, Foster City, CA); *RNU6B* or *GAPDH* were used as internal controls. The threshold cycle (Ct) method was used to quantitate the changes of expression [5].

### Western blot analysis

The Western blot analysis and the quantification of protein expression followed protocols that have been previously described [5]. The primary antibodies are listed in Supplementary Table S3. The anti-rabbit, anti-mouse and anti-goat secondary antibodies were obtained from Chemicon Int. (Billerica, MA).

### Cell culture

SAS, OECM1, FaDu and HSC3 HNSCC cells, 293T cell as well as a h*TERT* immortalized normal oral keratinocyte (NOK) cells, were cultivated according to previous protocols [4, 5]. *miR-31* mimic and inhibitor along with appropriate controls were purchased from Applied Biosystems. The siARID1A (L-017263-00-0005) and siEpCAM (L-004568-01-0005) oligonucleotides, together with scramble controls, were purchased from Dharmacon (Lafayette, CO). The optimized dose for both siARID1A and siEpCAM was identified as 60 nM for 48 h, and this was used during all experiments. Other chemicals were purchased from Sigma-Aldrich (St. Louise, MO).

### Plasmids

The pcDNA6-ARID1A vector was purchased from Addgene (Cambridge, MA). A vector carrying green fluorescence gene (GFP) was used to measure transfection efficiency [48]. The short hairpin shARID1A constructs (Supplementary Table S4) packed in lentiviruses were purchased from the RNA Interference Consortium (Academia Sinica, Taipei, Taiwan). After selection, cell subclones exhibiting knockdown of ARID1A were identified and designated as shARID1A 9090 and 9091. These were then used in the experiments together with shLuc subclone as a control.

### Phenotypic analysis

The phenotypes of the various cell lines, including proliferation, migration, invasion, anchorage-independent growth (AIG), ALDEFLUOR assay, sorted CD44^+^ cell population and spheroid formation assay followed protocols previously published [4, 30, 44]. SAS cells were injected subcutaneously into the flank (10^6^ cells) orthotopically into the central part of the tongue (1.5 × 10^5^ cells) of BALB/c athymic mice [4, 8]. After sacrifice of animal, the subcutaneous xenografts, the resected tongues and the dissected neck tissues were subjected to image analysis, processing and histopathological evaluation. The animal studies were performed according to the guidelines from the Institutional Animal Care and Use Committee (IACUC) of National Yang-Ming University and had approval No. 1021257.

### Reporter constructs and activity assay

The full-length of the 3′UTR sequence of the *ARID1A* gene was cloned into the pMIR-REPORTER plasmid (Applied Biosystems) in order to generate the pMIR-ARID1A-Wt construct. A mutant reporter construct (pMIR-ARID1A-Mut) was obtained from the Wt construct by replacing the original CUUGCC sequence at the target site with GAGCUC in order to create a new *Sac* I restriction enzyme digestion site; this was done using a PCR-based strategy (Supplementary Table S5). A SNP reporter was generated from Wt reporter by carrying out a change at nucleotide 999 from T→C.

The genomic regions flanking the promoter region of human Nanog (-382 ~ +22 to ATG), OCT4 (-264 ~ +470 to ATG), Sox2 (-484 ~ +52 to ATG) and EpCAM (-472 ~ +49 to ATG) were amplified by PCR (Supplementary Table S5) and inserted into the KpnI/HindIII sites of pGL3 vector (Promega, Madison, WI); this generated a series of wild type promoter reporter constructs, which were designated a WT promoters. Next, promoter constructs containing truncations of the various AT-rich sites, designated Del, were generated from the WT promoters by site-directed mutagenesis (Supplementary Table S5). The plasmid pRL-TL, which expresses the renilla luciferase gene, was co-transfected with the plasmids used in the experiments as a control for the transfection efficiency.

### Prediction of AT-Rich binding sites

Genomatix (https://www.genomatix.de/), an open-access prediction system [49], was used to predict AT-Rich binding sites in the promoter regions of various genes.

### ChIP assay

ChIP experiments were carried out using methods that we have established previously [50]. Sonicated chromatin was immunoprecipitated using anti-ARID1A antibody (Supplementary Table S3). The PCR reaction generated 168-bp, 239-bp, 154-bp and 234-bp amplicons of the proximal regions of the promoters of Nanog, OCT4, Sox2 and EpCAM containing AT-rich sites, respectively (Supplementary Table S5). The amplicons were separated on an agarose gel and then visualized. SYBR^®^ Green quantitative (q)PCR analysis was also performed.

### ISH analysis

The hsa-*miR-31*, the scrambled oligonucleotide control and various other reagents were purchased from Exiqon (Copenhagen, Denmark). Tissue sections from the HNSCC TMA were fixed and subjected to ISH analysis using the protocols that we have described previously [5]. The staining was captured by Image-Pro software (Media Cybernetic, Rockville, MD) and quantified using Photoshop software (Adobe; San Jose, CA) [5].

### IHC analysis

Tissue sections from the HNSCC TMA were processed for IHC using the protocols we have described previously [5]. The antibodies used are described in Supplementary Table S3. The immunoreactivity levels were captured and analyzed using the same methods as those used for ISH. Nuclear ARID1A immunoreactivity was scored based on the percentage of ARID1A positive nuclei as a proportion of the total cells counted.

### Statistical analysis

The data are shown as Means ± S.E. Mann-Whitney tests, *t*-tests, two-way ANOVAs, linear regression and Kaplan-Meier survival analysis were used to compare the differences between variants. The diagnostic power was analyzed by Receiver Operating Characteristic (ROC) curve. *ns*, not significant; *, *p* < 0.05; **, *p*<0.01; ***, *p*<0.001.

## SUPPLEMENTARY MATERIALS FIGURES AND TABLES




