# Supplementary material for: miR-31 targets ARID1A and enhances the oncogenicity and stemness of head and neck squamous cell carcinoma
Source: Oncotarget. 2016 Aug 9;7(35):57254–67. doi: 10.18632/oncotarget.11138 (PMC5302987; doi:10.18632/oncotarget.11138)
Supplement: Supplementary file 1 [file oncotarget-07-57254-s001.pdf]

miR-31 targets ARID1A and enhances the oncogenicity and stemness of head and neck squamous cell carcinoma

SUPPLEMENTARY FIGURES AND TABLES

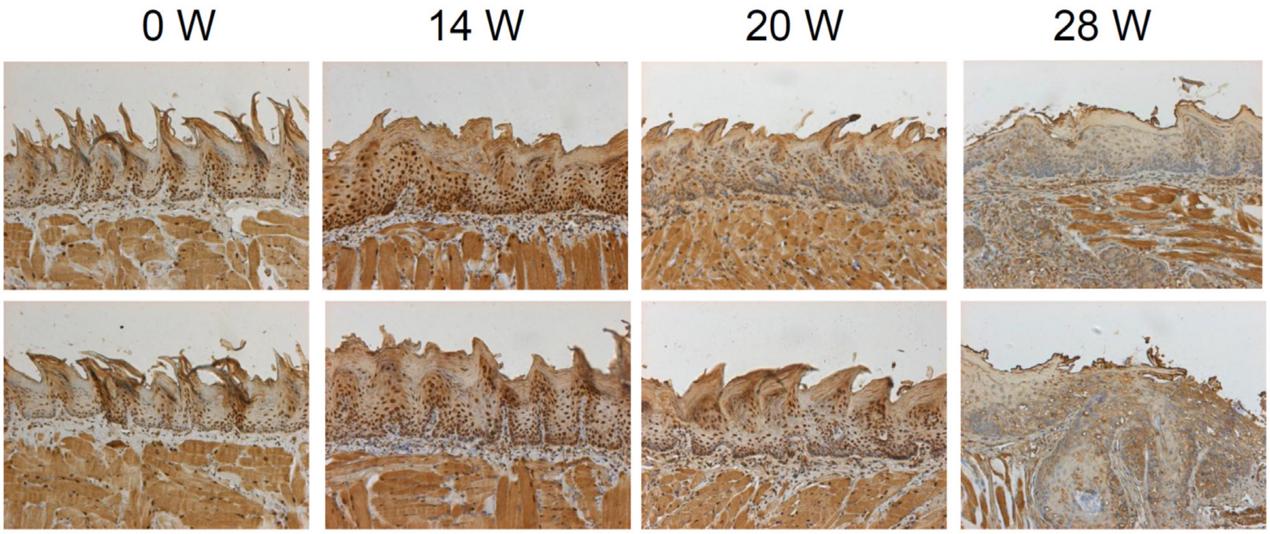

Supplementary Figure S1: ARID1A immunoreactivity in mouse multistep carcinogenesis. Mouse tongue tissues upon 4NQO treatment for 0, 14, 20 and 28 weeks. (x100). Upper panels and lower panels are from different mice. Except for the Rt Lowest panel, which is an invasive tumor, all other panels are normal looking mucosa. Note the nuclear and cytosolic immunoreactivity in epithelial cells. The nuclear ARID1A immunoreactivity scored as percentage are summarized in Supplementary Figure 1A, Rt.

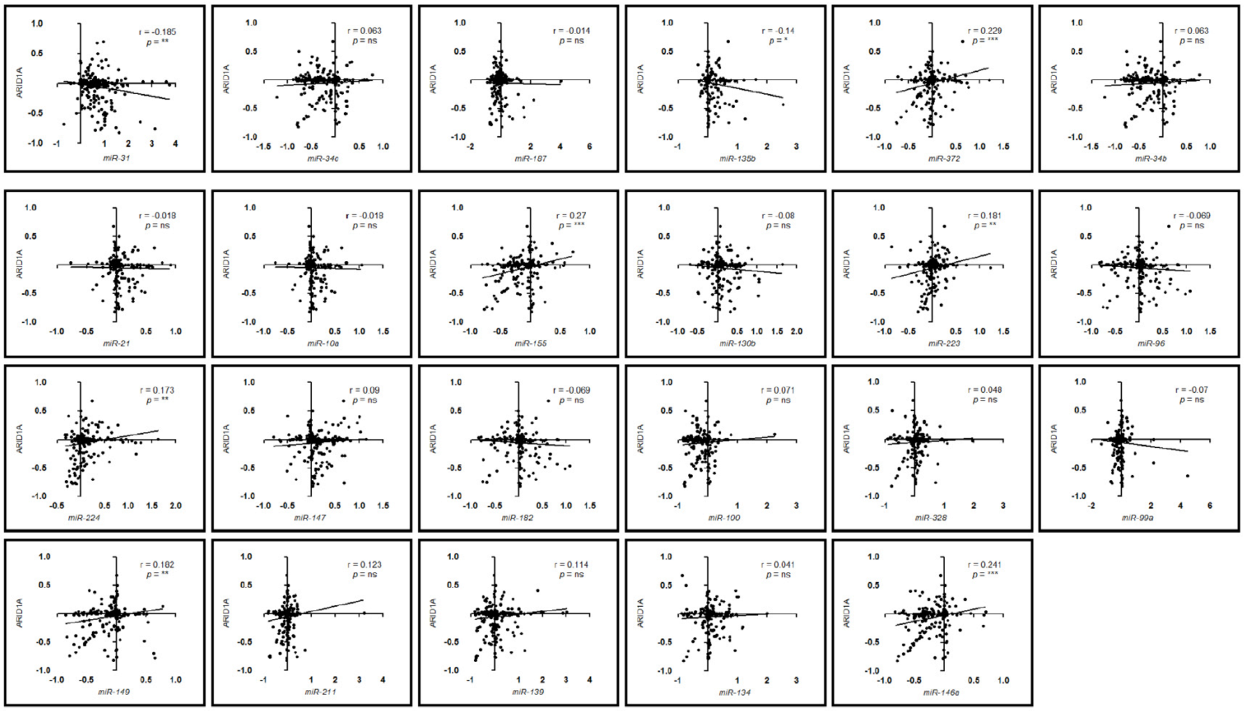

Supplementary Figure S2: Correlation between the expression of oncogenic miRNAs and *ARID1A* mRNA expression in HNSCC TCGA database. The algorithm of *r* values and the reverse correlation noted between *miR-31* and ARID1A are shown in Supplementary Figure 1E.

	miRWalk	miRanda	mirbridge	miRDB	miRNAMap	Pictar2	PITA	RNA22	Targetscan	DIANA
hsa-*miR-31*	+	+	+	+	+	+	+	+	+	+
hsa-*miR-135b*	-	-	-	-	-	-	-	+	-	-

Supplementary Figure S3: Prediction of potential *miR-31* and *miR-135b* binding site in the 3'UTR of ARID1A gene. +, positive for a predicted binding; -, negative for a predicted binding.

A

Cell	ARID1A 3’UTR
OECM1, HSC3, FaDu, 293T, NOK	ATTTAATCTCTTGCCAGATATCGCCCC
SAS	ATTTAATCTCTTGCCAGA[T/C]ATCGCCCC

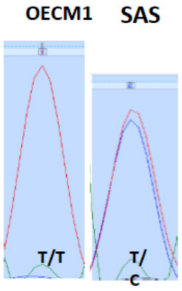

B

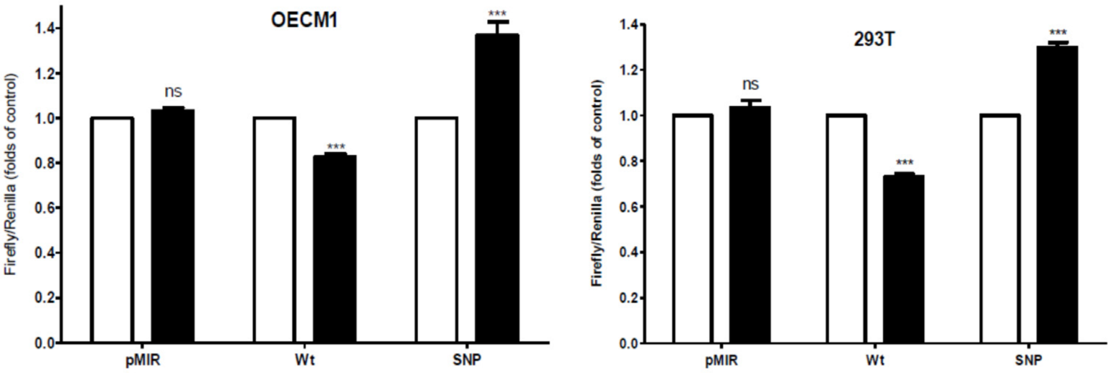

Supplementary Figure S4: rs12685 polymorphism within ARID1A 3’UTR of SAS cells. A. Lt, Summary of the 3’UTR sequence of ARID1A in various oral keratinocytes and 293T cells. Rt, Sequencing analysis reveals the presence of a heterozygous T/C sequence at nucleotide 999 within SAS cells. **B.** Assays of Wt and SNP reporters after treatment of *miR-31* mimic in OECM1 and 293T cells.

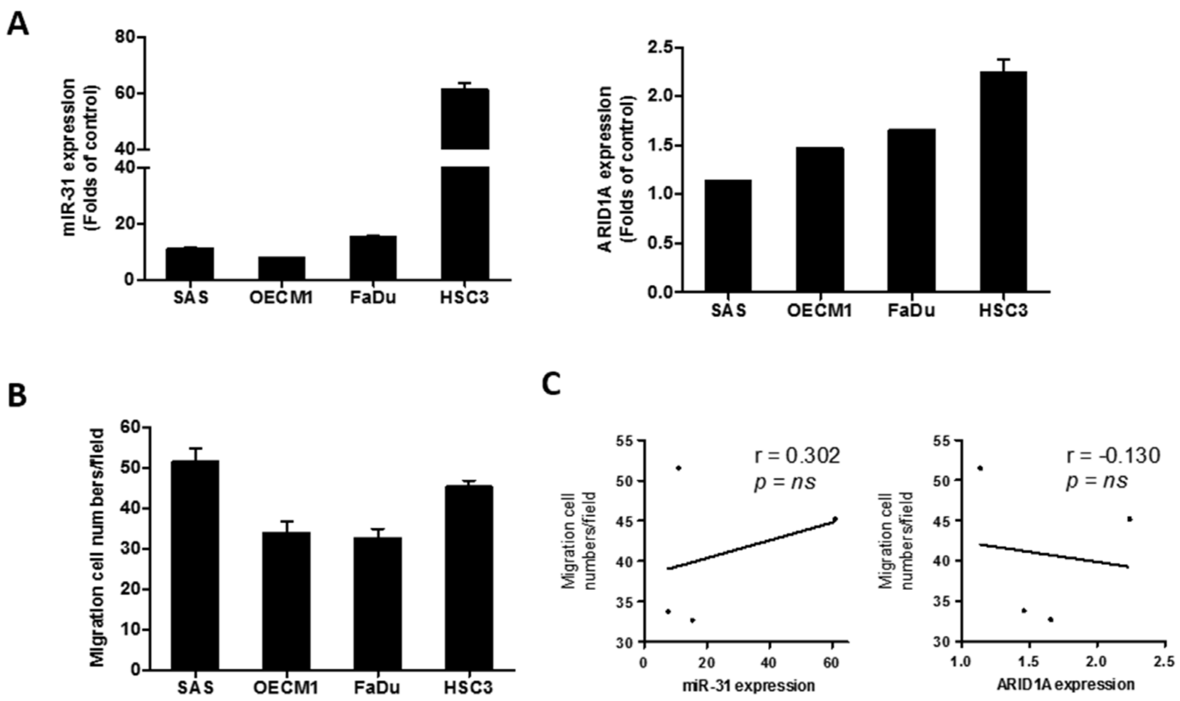

Supplementary Figure S5: Correlation between the expression of *miR-31* and ARID1A and the migration ability in four HNSCC cell lines. A. Endogenous *miR-31* expression (Lt) and ARID1A (Rt) expression. NOK is the control. **B.** Migration analysis. **C.** Linear regression analysis shows no correlation between cell migration and the expression level of *miR-31* (Lt) or ARID1A (Rt).

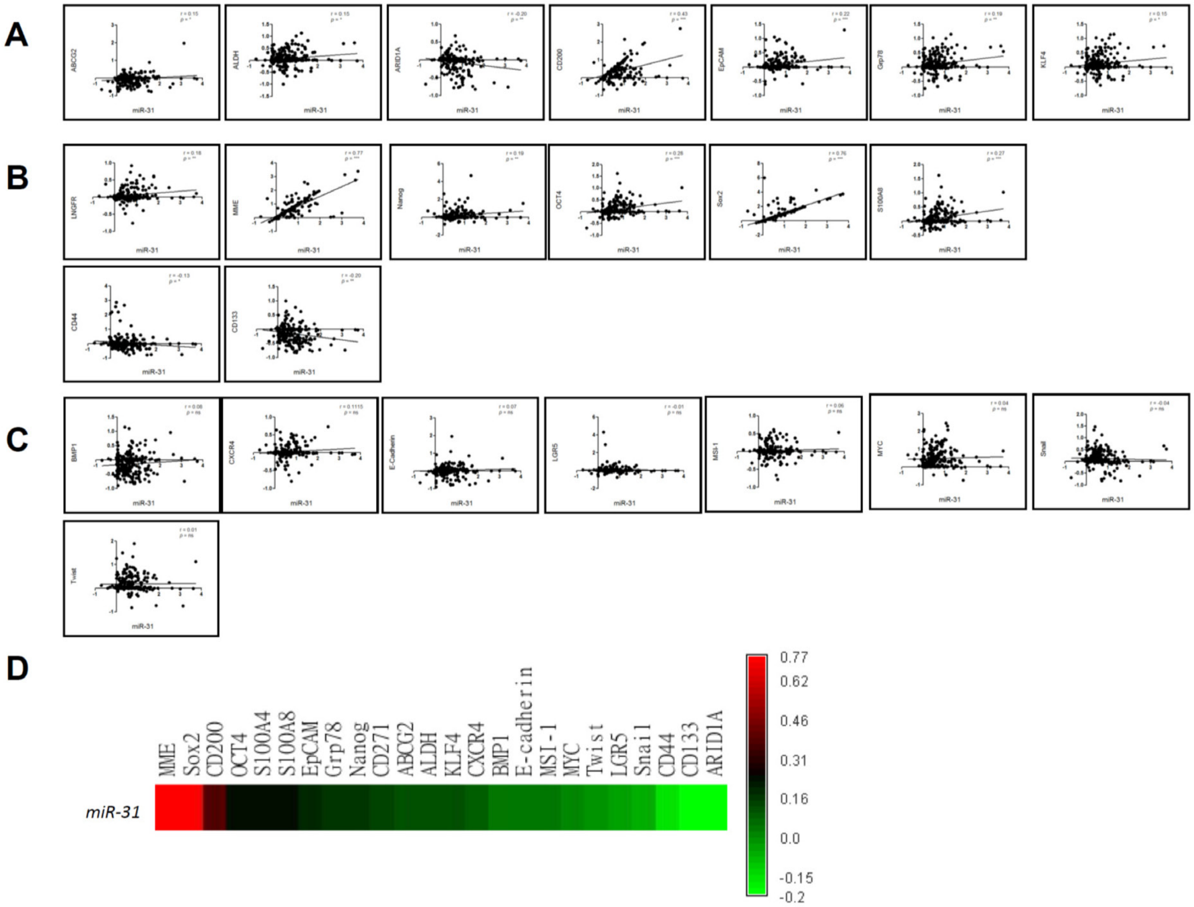

Supplementary Figure S6: Correlation between the expression of stemness related genes and *miR-31* expression in HNSCC TCGA database. A. Genes with positive correlation. **B.** Genes with reverse correlation. **C.** Genes without correlation. **D.** An algorithm illustrating the *r* values of genes analyzed.

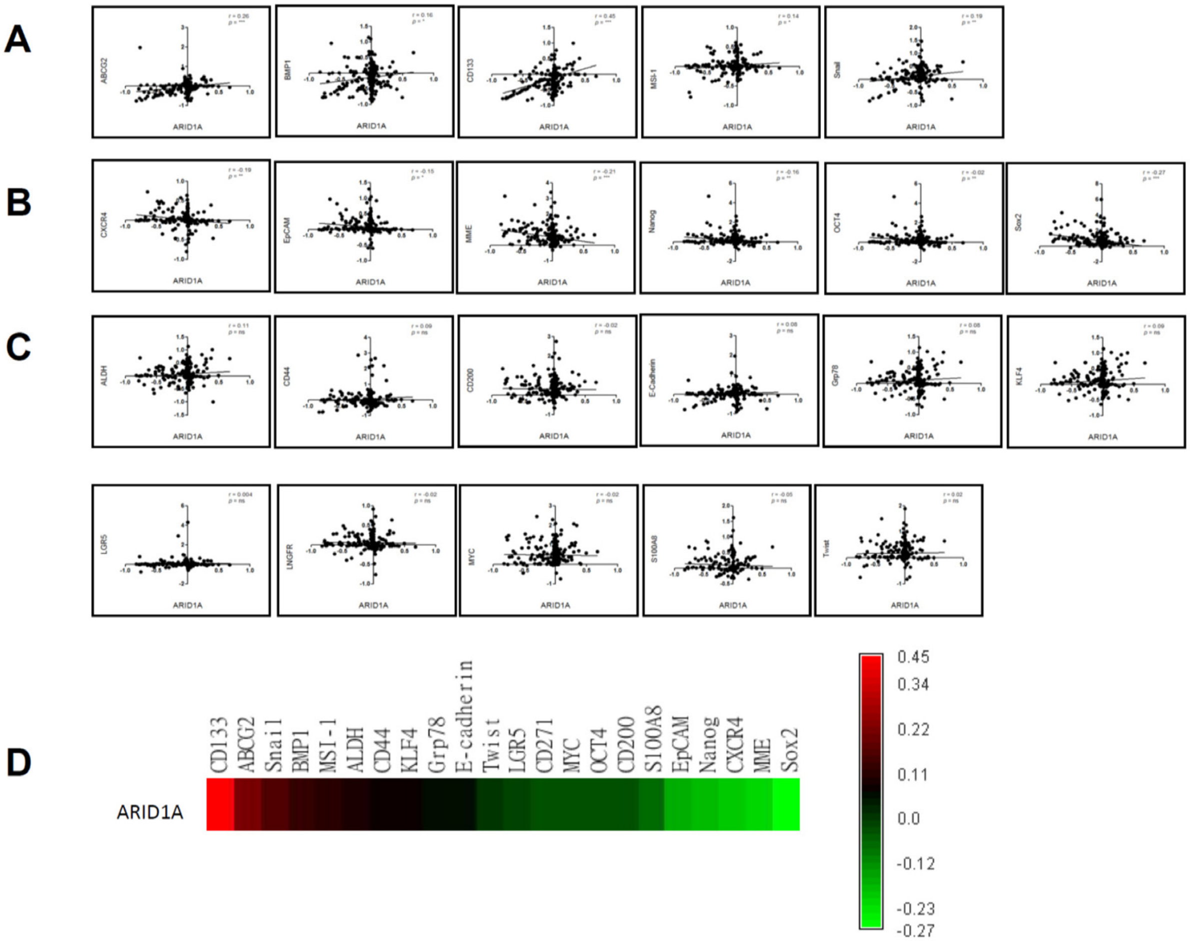

Supplementary Figure S7: Correlation between the expression of stemness related genes and *ARID1A* expression in HNSCC TCGA database. A. Genes with positive correlation. **B.** Genes with reverse correlation. **C.** Genes without correlation. **D.** An algorithm illustrating the *r* values of genes analyzed.

AT-Rich Site Prediction

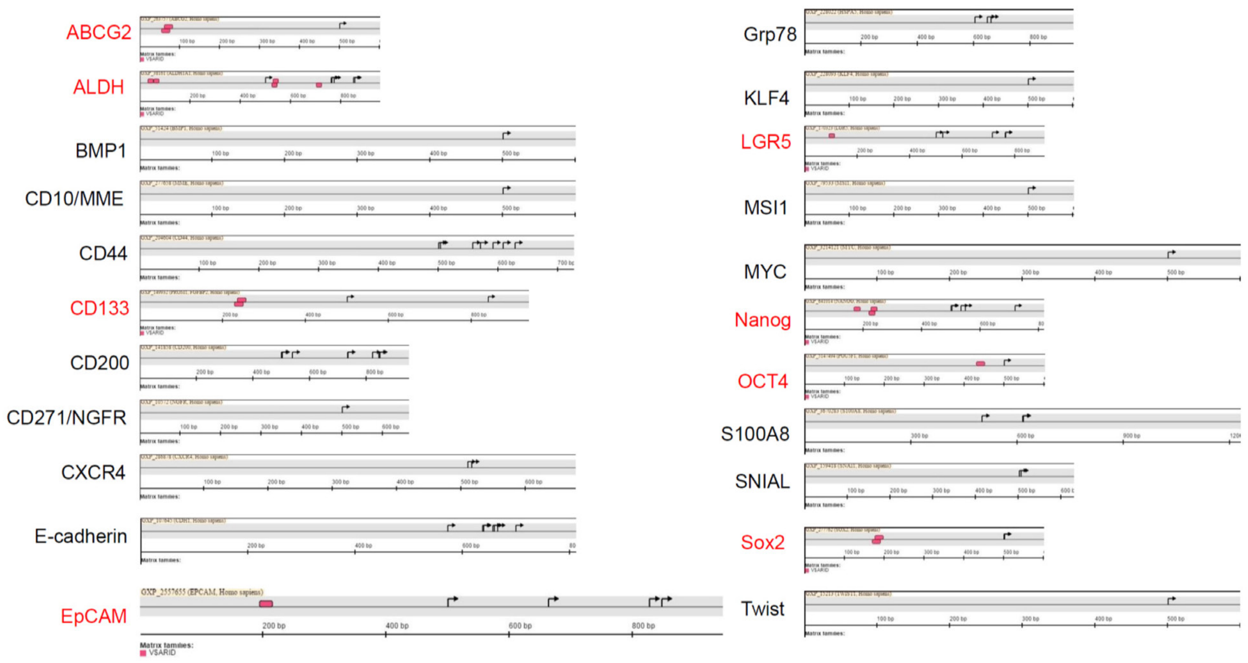

Supplementary Figure S8: Prediction of AT-rich sites in promoters. The prediction map of AT-rich binding site in -1000-TSS region in promoters of all genes. Predicted sites are marked in red boxes. Predicted genes are marked with red fonts.

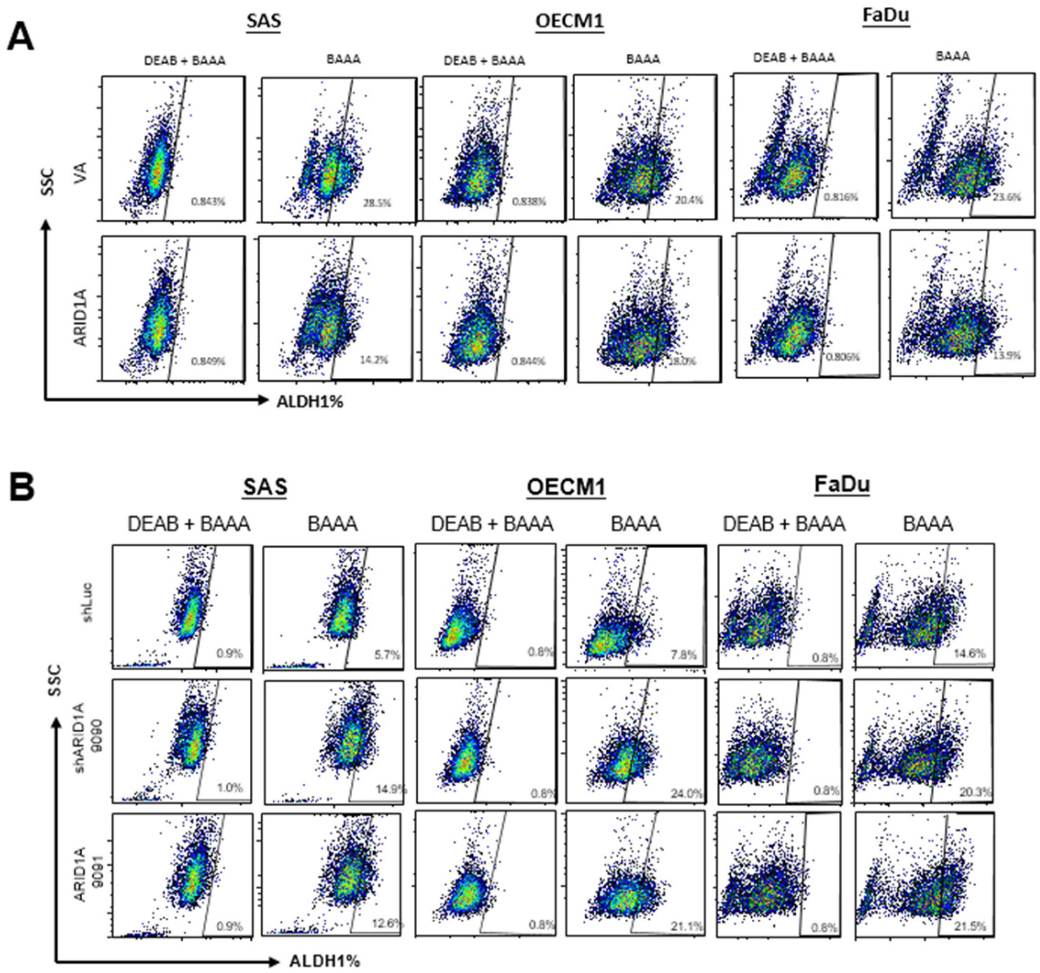

Supplementary Figure S9: ARID1A suppresses stemness property in HNSCC cells. A, B. The ALDEFLUOR assay. An increase or a decrease in the cell population with ALDH1 activity (ALDH1^{+}) is noted for the SAS, OECM1 and FaDu cell subclones with ARID1A overexpression (in A) or knockdown (in B). The percentages are presented in the pictures.

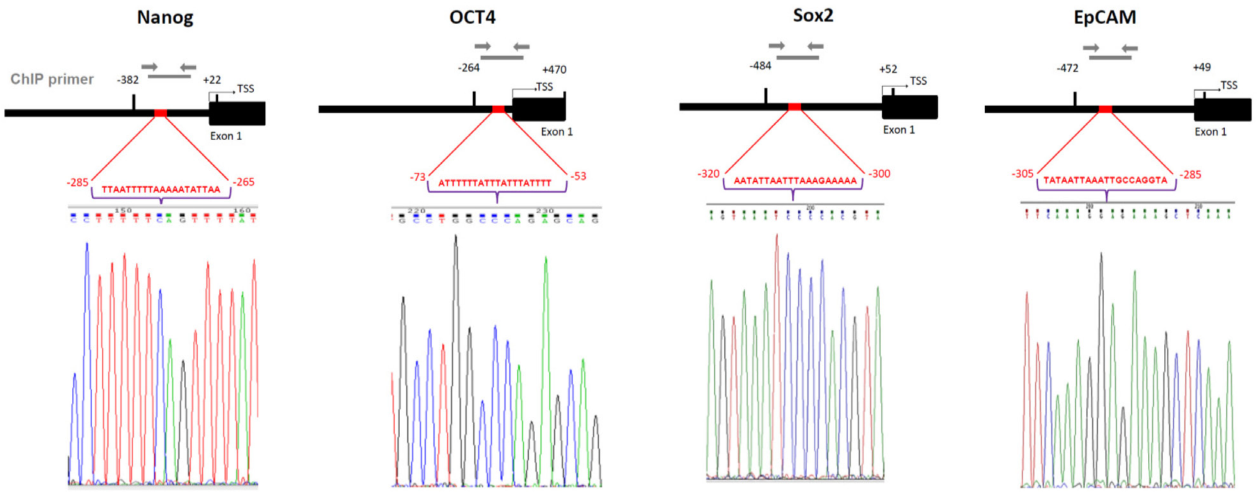

Supplementary Figure S10: Prediction of AT-rich sites in Nanog/OCT4/Sox2/EpCAM promoter region. Detailed diagrams designate AT-rich sites (Red boxes) in the proximal regions of the Nanog, OCT4, Sox2 and EpCAM promoters. TSS, transcription start site; Red bars, the termini of AT-rich sites. Black bars define the segments for reporter assay. Grey lines define the segments for ChIP assay. Bottom, the sequencing analysis demonstrated the deletion of AT-rich sites in Del reporter constructs.

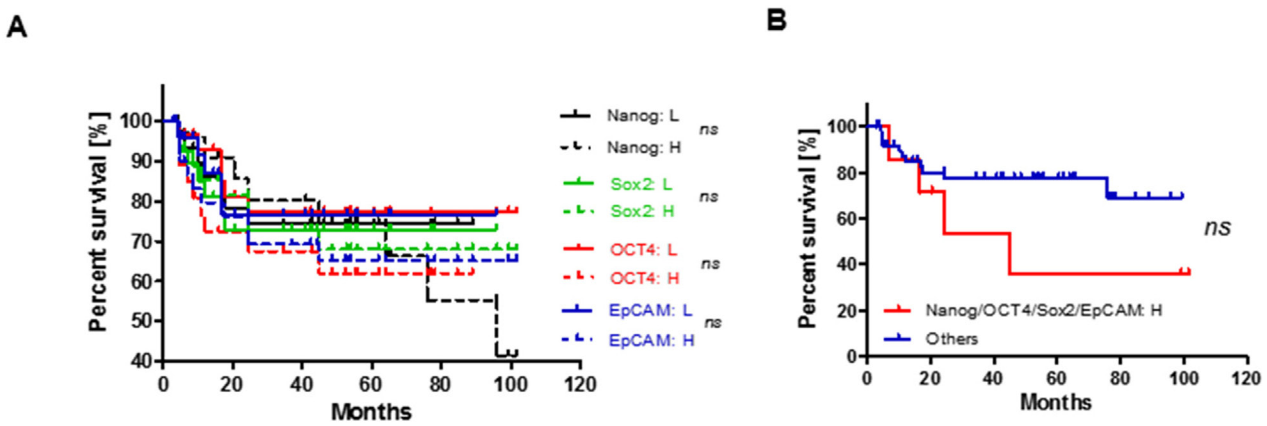

Supplementary Figure S11: Disease free survival. A. Analysis according to the expression of solitary pluripotency factor in tumor. **B.** Analysis according to the expression of all pluripotency factors in tumor. H, high expression; L, low expression.

Supplementary Table S1: Clinicopathological parameters of paired OSCC for Western blot and qRT-PCR analysis

n **=**	**58**
Age (Mean ± SE years)	56.9 ± 1.4
Gender (Male/Female)	52/6
TNM staging	
T1-3	24
T4	34
N0	38
N+	20
Stage I	8
Stage II	9
Stage III	7
Stage IV	34

Supplementary Table S2: Clinicopathological parameters of OSCC TMA for IHC and ISH analysis

***n* =**	**60**
Age (Mean ± SE years)	54.7 ± 1.6
Gender (Male/Female)	52/8
TNM staging	
T1-3	17
T4	43
N0	42
N+	18
Stage I	4
Stage II	7
Stage III	6
Stage IV	43

Supplementary Table S3: Primary antibodies used in the present study

Antibody	MW (kDa)	Host	Dilution	Supplier	Cat. No.
ARID1A	240	Rabbit	1:3000	Sigma-Aldrich	HPA005456
ARID1A*	240	Rabbit	1:200	Sigma-Aldrich	HPA005456
ARID1A#	240	Mouse	1:200	Santa Cruz Biotech	SC-32761X
Nanog	42/44	Rabbit	1:1000	Cell Signaling	3580
FIH	40	Goat	1:1000	Santa Cruz Biotech	SC-26219
Nanog*	35	Rabbit	1:100	Abcam	Ab109250
OCT4	42/44	Rabbit	1:1000	Cell Signaling	2750
OCT4*	42/44	Rabbit	1:200	Cell Signaling	2750
Sox2	36	Rabbit	1:1000	Cell Signaling	3579
Sox2*	36	Rabbit	1:200	Cell Signaling	3579
KLF4	52	Rabbit	1:1000	Abcam	151733
GFP	27	Mouse	1: 5000	Clontech Lab	632459
Grp78	78	Mouse	1:1000	BD Biosciences	610979
EpCAM	35	Rabbit	1:1000	Abcam	71916
EpCAM*	35	Rabbit	1:160	Abcam	71916
GAPDH	36	Mouse	1:10000	Santa Cruz Biotech	SC-32233

*For IHC analysis.
#For ChIP assay.

Supplementary Table S4: shRNA utilized in the present study

Symbol	Clone ID	Clone Name	Vector Name	Sequence
shLuc	TRCN0000072249	promegaLuc_976s1c1	pLKO.1	GCGGTTGCCAAGAGGTTCCAT
ARID1A	TRCN0000059090	NM_006015.3-1702s1c1	pLKO.1	CCTCTCTTATACACAGCAGAT
ARID1A	TRCN0000059091	NM_006015.3-7163s1c1	pLKO.1	CCGTTGATGAACTCATTGGTT

Supplementary Table S5: Primers used in the present study

See Supplementary File 1
