# Supplementary material for: miR-31 targets ARID1A and enhances the oncogenicity and stemness of head and neck squamous cell carcinoma
Source: Oncotarget. 2016 Aug 9;7(35):57254–67. doi: 10.18632/oncotarget.11138 (PMC5302987; doi:10.18632/oncotarget.11138)
Supplement: Supplementary file 2 [file oncotarget-07-57254-s002.docx]

**Table S5. Primers used in the present study**

| **Gene or Construct** | **Forward primer (5’-3’)** | **Reverse primer (3’-5’)** | **Amplicon (bps)** |
| --- | --- | --- | --- |
| ARID1A  Wt reporter | GGGACTAGTCTACGCTGCCACGTGTGTAT | GGGAAGCTTTTCATCAGAACTTCAACTGAACC | 324 |
| ARID1A  Mut reporter | CACAGCTATTTAATCGGAGCTCGGATATCGCCCCTCTT | AAGAGGGGCGATATCCGAGCTCCGATTAAATAGCTGTG | 324 |
| Nanog WT promoter | CTAGCAAAATAGGCTGTCCC | GACGATAGTCATGCCCCGCG | 404 |
| Nanog Del promoter | CGCGCCCGGCCTTTTTCAGTTTTATCCCATTCCTG | GGAATGGGATAAAACTGAAAAAGGCCGGGCGCG | 384 |
| OCT4 WT promoter | GGGGGTACCGCCCGGACTGCTTTACTTTT | GGGAAGCTTTCTCTCACTCAAGTATCACCCC | 734 |
| OCT4 Del promoter | CACTGTGCCTGGCCCAGAGCAGGAGTGG | CCACTCCTGCTCTGGGCCAGGCACAGTG | 714 |
| Sox2 WT promoter | GGGGGTACCATGAGCGGGAGAACAATGAC | GGGAAGCTTCTGCCTTGACAACTCCTGAT | 536 |
| Sox2 Del promoter | ACCCTGCACCAAAAAGTAAATCCCCACGTAGTCTTAG | CTAAGACTACGTGGGGATTTACTTTTTGGTGCAGGGT | 516 |
| EpCAM WT promoter | GGGGGTACCAGCCTGGGAACACCTTTTCT | GGGAAGCTTAGTTGGGGGAGTGAGTAGGC | 521 |
| EpCAM Del promoter | AGAACACTATAAAAAAGACCTTTGAGCTTTCTCCTTTGAAATTAGAAAGAAAAAAAAAAG | CTTTTTTTTTTCTTTCTAATTTCAAAGGAGAAAGCTCAAAGGTCTTTTTTATAGTGTTCT | 501 |
| Nanog-ChIP | CACGGCCTCCCAATTTACTG | TGAAGACAAACCCAGCAACA | 168 |
| OCT4-ChIP | TGTTACCCAGGCTGCTTTCT | GCTATTCGGCACTTGTTCTTCA | 239 |
| Sox2-ChIP | CCGCGTCCCATCCTCATTTA | ACCTTTGTATCCCCTCTCGC | 154 |
| EpCAM-ChIP | AGCCTGGGAACACCTTTTCT | ACAGGCAGAGAACCTTCCAG | 234 |
